# Fabrication of crystalline submicro-to-nano carbon wire for achieving high current density and ultrastable current

**DOI:** 10.1038/s41378-021-00345-z

**Published:** 2022-02-04

**Authors:** Jufeng Deng, Chong Liu, Dian song, Marc Madou

**Affiliations:** 1 School of Mechanical Engineering, Dalian University of Technology, Dalian, 116023 China; 2 Mechanical and Aerospace Engineering, University of California, Irvine, CA 92617 USA; 3 Chemical and Biomolecular Engineering, University of California, Irvine, CA 92517 USA; 4 School of Engineering and Science, Tecnologico de Monterrey, Monterrey, NM 64849 Mexico

**Keywords:** NEMS, Nanowires

## Abstract

Crystalline carbon nanowire arrays were fabricated taking advantage of near-field electrospinning and stress decyanation. A novel fabrication method for carbon nanowires with radii ranging from ~2.15 µm down to ~25 nm was developed based on implementing nitrogen pretreatment on the silica surface and then aligning polymer nanofibers during near-field electrospinning at an ultralow voltage. Stress decyanation was implemented by subsequently pyrolyzing a polymer nanofiber array on the silica surface at 1000 °C for 1 h in an N_2_ atmosphere, thus obtaining a crystalline carbon nanowire array with a nanostructured surface. Various crystalline nanostructures were fabricated on the nanowire surface, and their electrochemical performance was evaluated by cyclic voltammetry (CV) and electrochemical impedance spectroscopy (EIS). Crystalline carbon wires with diameters ranging from micrometers to submicrometers displayed carbon nanoelectrode-like behavior with their CV curve having a sigmoidal shape. A highly crystalline carbon nanowire array showed distinct behavior, having a monotonically increasing straight line as its CV curve and a semicircular EIS spectrum; these results demonstrated its ultrastable current, as determined by electron transfer. Furthermore, nanocrystalline-structured carbon wires with diameters of ~305 nm displayed at least a fourfold higher peak current density during CV (4000 mA/m^2^) than highly crystalline carbon nanowires with diameters of ~100 nm and porous microwires with diameters of ~4.3 µm.

## Introduction

The attractive merit of carbon wires derives from carbon microstructures integrating high-performance with abundant functionalities^1,2^. There are several available microstructures of carbon materials, such as glassy carbon, diamond, and graphite. Carbon nanowires with glassy carbon structures have been used in many different applications, such as in high-power supercapacitors^3^, electrochemical biosensors^4^ and high-energy rechargeable batteries^5^. To upgrade all of the intrinsic properties in the case of conventional carbon nanowires with glassy carbon structures, the principal philosophy emphasizes the interconnected graphitic structure, which provides an extraordinary combination of mechanical, electrical, and thermal properties (strength^6^ up to 20 GPa, electrical conductivity^6^ of 1.5 × 10^6^ S/m and thermal conductivity^7^ of 5000 W/m·K). Compared to glassy carbon nanowire configurations, graphitized carbon nanowires offer several advantages, such as their extraordinary mechanical properties, which includes their elastic modulus^8^ (1.1 TPa), tensile strength^8^ (~130 GPa) and notable flexibility, and their excellent electron transport performance^9^, which includes their extremely high electric conductivity (~10^8^ S/m), carrier mobility (2000000 cm^2^/V·s) and ampacity (1–2 GA/cm^2^). These advantages demonstrate the abundant functionalities of graphitized carbon nanowires for use in various applications, such as in electrical conductors^10^, supercapacitors^11^, actuators, and solar devices^12^, showing more applicability than conventional carbon nanowires. Regarding electrochemical energy storage, graphitized carbon nanowires are promising for enhancing energy and power densities.

The thermal reduction and high-temperature annealing of graphite oxide wire made by a wet-spinning process have been proposed to produce highly graphitized carbon wires but are limited to micron-scale fabrication^13^. For the electrochemical applications mentioned above, the performance is typically improved by increasing the surface area of graphitized carbon nanowires, which can be achieved by decreasing the diameter of graphitized carbon nanowires. Most of the techniques that have been proposed for fabricating graphitized nanoscale carbon wires include mechanical stress pyrolysis^14^ and chemical vapor deposition^15^. The doping approaches involved in these techniques cause additional substances to be added into the carbon wires, which affects the electrochemical behavior of the carbon wire. This has led to extensive research into the pyrolysis of near-field electrospun fibers (PNFEFs), which enables advanced applications in electrochemical sensing, energy storage, and stem cells.

PNFEFs are dedicated to manufacturing new classes of well-defined carbon nanostructures. In this process, polymer nanofibers are arrayed by the near-field electrospinning of a precursor solution and subsequently converted into pyrolytic carbon nanowires through high-temperature treatment (>800 °C) in an inert atmosphere. This method offers excellent control of feature sizes, density per unit area, and the capability to pattern carbon nanowires. By introducing carbon scaffolds into this process, a dramatic decrease in the fiber diameter leads to ultrathin carbon nanowires (~5 nm)^16^. This technique is mostly established because the high-throughput fabrication of carbon nanowires can be easily achieved.

Recently, highly graphitized carbon nanowires^17^ have been developed on carbon scaffolds using near-field electrospinning, pyrolysis, electrodeposition, and chemical vapor deposition. Clearly, these techniques are still inseparable from the participation of nickel catalysts, resulting in an inability to remove the nickel from the carbon wire. Thus, to date, the electrochemical behavior of highly crystalline carbon nanowires with graphitic structures have not been improved. Here, we present the fabrication and characterization of highly crystalline carbon nanowire (HCCN) arrays with well-controlled wire-to-wire spacing. For this purpose, a novel method for the fabrication of highly crystalline carbon nanowires is implemented, permitting control of the wire diameter, wire-to-wire spacing, and degree of graphitization. By the programmed movement of a linear stage during near-field electrospinning (NFES), the polymer nanofibers are arrayed on a silicon substrate coated with silica (500 nm thickness). Before depositing the polymer nanofiber arrays, the silica surface deposited by polymer fibers is pretreated at 1000 °C in a nitrogen atmosphere. To convert the polymer fibers into carbon nanowires, a silicon chip with carbon dioxide and polymer fibers is pyrolyzed at 1000 °C in a nitrogen atmosphere. The microstructure of the carbon wires is evaluated by atomic force microscopy and Raman spectroscopy. Additionally, the fabricated HCCNs are characterized electrochemically with cyclic voltammetry and electrochemical impedance spectroscopy (ESI).

## Results and discussion

### Fabrication of polymer nanofiber arrays using near-field electrospinning (NFES)

**Fig. 1:**
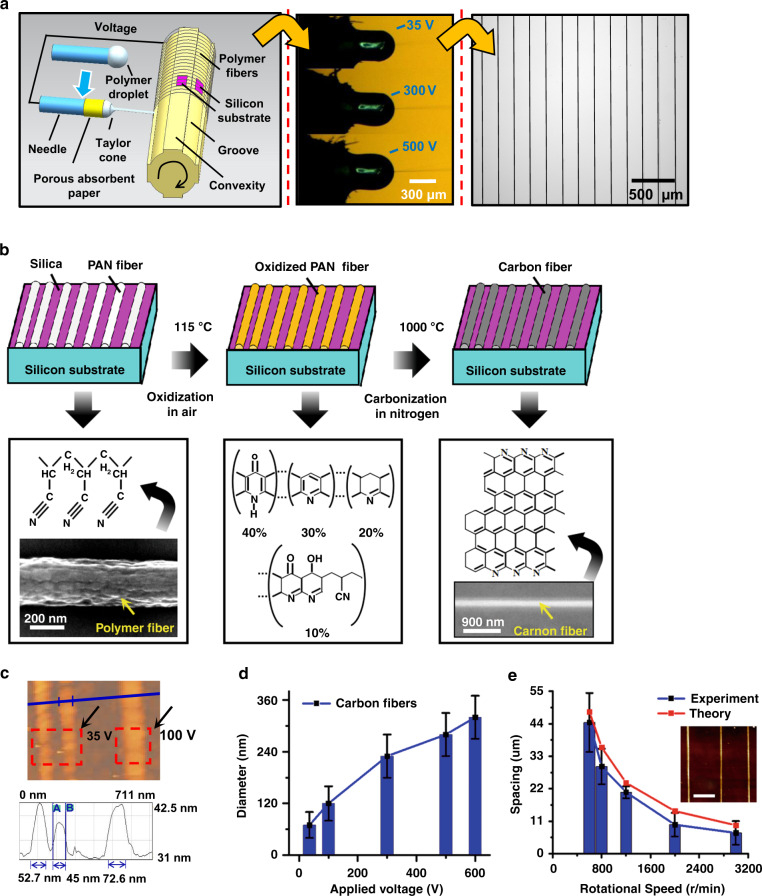
Nanoscale carbon wires from PAN fibers. **a** Thin PAN nanofiber array on a silicon substrate coated with silicon dioxide: continuous deposition of NFES PAN nanofibers on substrates mounted on a rotating drum and electric jet ejection at different applied voltages from 500 to 35 V. **b** Fabrication of carbon nanowires from PAN fibers at 300 V. **c** Atomic force microscopy (AFM) images of the carbon nanowires at 100 and 35 V. **d** Carbon wire diameter as a function of applied voltage; *n* > 30. **e** Highly uniform spacing between the carbon wires in an array obtained by varying the rotational speed at a constant low voltage of 35 V; *n* > 50

To attain the thinnest polymer fibers at the lowest applied voltages in NFES, one important procedure concerns the protocol for preparing the ink by the dissolution of polyacrylonitrile (PAN) in *N*,*N*-dimethylformamide (DMF). A heat treatment from 60 to 126 °C is found to be accompanied by a conductivity change, which indicates the highest conductivity at 106 °C^16^. The conductivity data can be collected by a conductivity meter (OAKTON CON 510 Series) with the ability to show both conductivity and solution temperature. The conductivity plays a role in inducing sufficiently large electrical stress, which makes it possible to initiate a nano jet during near-field electrospinning. With a drum-to-needle distance of 5 mm, the polymer jet does not initiate even at 1500 V because the electrostatic force cannot overcome the surface tension at the droplet-air interface. However, touching the ink droplet at the ejector needle tip with the rotating drum causes the electrical stress to become large enough to counterbalance the surface tension stress, giving rise to the formation of a Taylor cone and jet initiation at a voltage as low as 500 V (see Fig. 1a). When using a PAN ink heated to either 60 °C or 126 °C, jet initiation at 500 V is not achieved by the same touching procedure, which demonstrates the key role of conductivity for initiating the jet during NFES.

A porous absorbent paper mounted around the base of the ejector needle thins the liquid layer around the needle at low feed rates (1 nL/min). The resulting electric field intensity at the liquid-air interface is substantially higher than that of the nozzle without droplet shaping at the same voltage^18^. This in turn results in the decrease in the applied voltage from 500 to 35 V, which is far below any current low-voltage NFES practice (see Fig. 1a). During NFES, continuous jetting at an ultralow applied voltage of 35 V becomes possible (see Fig. 1a). This reduction in the working voltage causes a decrease in the jet diameter, as described by Eq. (1):^16^

1 $$h = {\mathrm{InverseFunction}}\left\{ {\left[ { - \frac{{E^2K^2Inx}}{{8I^3}} + \frac{{E^2K^2In\left( { - 2I + EKx^2} \right)}}{{16I^3}} + \frac{1}{{8Ix^4}} + \frac{{EK}}{{8I^2x^2}}} \right]\left[ {\frac{{Ex}}{{2Q^3\sqrt \beta }} + C_1} \right]} \right\}$$

where *h* is the cross-sectional fiber radius, *E* is the electric field strength, *K* is the electric conductivity, *I* is the total current of the electrified jet, *x* is the distance between the needle and drum along the axis of the needle, *Q* is the volume flow rate, *β* = *ϵ*^3/2^-1 is the dimensionless conductivity of the fluid^19^, *ϵ* is the dielectric constant and *C*_1_ is a constant.

After the polymer jet is initiated at various voltages from 500 to 35 V, the alignment of polymer nanofibers on the silicon substrate is implemented by the programmed movement of a linear stage on which the needle is affixed (see Fig. 1a). In this automated positioning system, the rotational speed *ω* of a drum and the linear speed *ν* of the nozzle moving along the surface of that drum can readily tune the fiber-to-fiber spacing *d* as follows:

2 $$d = v/\omega$$

where *ν* is the linear speed of the nozzle moving along the surface of the drum, and *ω* is the rotational speed of the drum. In comparison with traditional electrospinning, shear force spinning during NFES affords a more uniform fiber-to-fiber spacing, as indicated by an ~2-fold decrease in the relative standard deviation of the achieved spacings^19^. Therefore, a rotating drum is introduced to add shear force into our electrospinning system, ultimately resulting in the more uniform alignment of polymer fibers shown in Fig. 1a. A comparison between the theoretical and experimental results is illustrated in Fig. 1e, revealing that maximizing the rotational speed (*ω*) and minimizing the linear stage speed (*ν*) makes it possible to lower the fiber spacing to as small as ~2.5 µm; thus, the density per unit area of the carbon nanowires on the silicon substrate can be maximized. Controlling the wire-to-wire spacing and carbon nanowire diameter makes it possible to obtain materials with higher sensitivity and stability for biosensing applications and kinetic studies^20^.

At a constant voltage of 35 V, polymer fibers in the convex parts of the drum are thinner than those in the grooved parts (see the right image in Fig. 1a). This is most likely due to the increase in the mechanical stretching of the nanofibers between the point of contact on the silicon substrate and droplet. By implementing a very low NFES working voltage of 35 V and programming the linear movement of the stage, polymer nanofibers with diameters as low as 50 nm diameter are aligned and deposited on silicon substrates fixed in the convex areas of the drum. The limitations of the proposed method lie in the dependency of initiating the jet and arraying fibers on the rotating drum. Instead of arraying fibers, making three-dimensional fiber scaffolds or films with this fabrication method has to undergo breaks in the lines due to the instability of the fluid flow inside the needle during long-term jetting.

Pyrolysis at 1000 °C leads to radial and longitudinal contraction as well as an enhanced tensile strength of the stabilized PAN nanofibers because of the increased carbon content^21–23^. As a result, a radial shrinkage of less than 15% in Fig. 1b is observed, which is lower than the 47.7 to 90% shrinkage^17^ from freely suspended polymeric nanofibers. This is most likely due to the strong attachment of the polymeric nanofiber to the silicon substrate, which derives from the impact of the polymer fiber on the silicon substrate during deposition. A combination of the effects of surface wetting and thermal expansion coefficient mismatch at the interface contributes to the possible interaction between polymeric nanofibers and a silicon substrate. The height of the carbon wire is much smaller than the width in Fig. 1c, showing fiber deformation caused by strong attachment. Due to lower shrinkage, the fabrication of a thin carbon wire largely depends on reducing the diameter of the polymer fiber. Ultralow-voltage (35 V) NFES allows for the smallest polymer fiber diameter, achieving thin carbon nanowires with diameters down to 45 nm, as shown in Fig. 1c. The effect of the applied voltage on the thickness of the carbon nanowires in Fig. 1d clearly reveals a decrease in the average diameter of the carbon nanowires with decreasing applied voltage during NFES. Since we initiate jetting with an ultralow voltage (35 V), the resulting carbon nanowires have an average diameter of 60 nanometers, which is far thinner than typical carbon nanowire diameters^24^. The dependency of the electrical properties on the size demonstrates that thinner carbon nanowires tend to far exceed the electrical properties of traditional carbon fibers^25^.

### Effect of aligning carbon nanowires and implementing a nitrogen pretreatment on the nanocrystalline structuring of carbon nanowires

Many reports conclude that the degree of a graphitic microstructure in carbon wires is related to the physical synthesis conditions, such as stress-induced routes^14^, torque-applied stretching^26^, and mechanical and electrostatic stretching^27^. A novel method for improving the graphitic microstructure of carbon wires on silica surfaces is developed by structuring nanocrystalline materials in this paper, which lays a foundation for studying the electrochemical properties of graphitic microstructures. To understand the mechanism of structuring nanocrystalline materials, disorderly carbon nanowires and partially aligned carbon wires are introduced as reference objects.

**Fig. 2:**
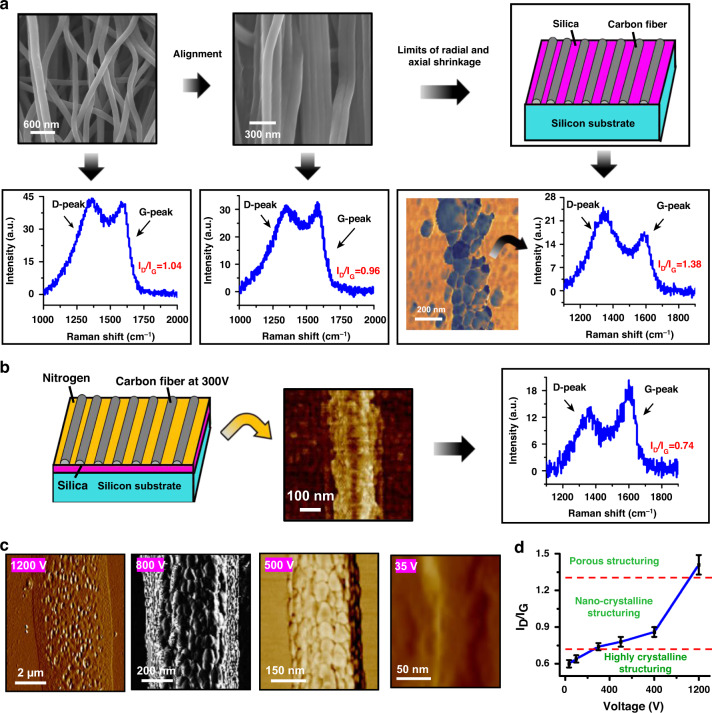
Effect of voltage-dependent nanowire size on the crystalline structure in regard to nitrogen pretreatment. **a** Limiting the radial and axial shrinkage to develop tensile stress. **b** Crystalline carbon nanowires on silica surfaces processed with nitrogen pretreatment at 300 V. **c** AFM phase images of carbon nanowires at 1200, 800, 500, and 35 V. **d** Transformation of the crystalline structure from porous to highly crystalline; *n* > 35

After jet initiation at 500 V in Fig. 1a, an increase in the applied voltage to 1200 V allows for the continuous electrospinning of a single, stable filament from a droplet at a flow rate of 1 nl/min. In this case, increasing the needle-drum distance from 1.85 mm to 10 mm during NFES results in the formation of disorderly polymer fibers and partially aligned polymer fibers onto the rotating drum. Subsequent stabilization in the air at 115 °C and then carbonization in a nitrogen atmosphere at 1000 °C (at a heating rate of 15 °C/min from 260 to 1000 °C) transform such polymer fibers into carbon nanowires, as shown in Fig. 2a. In general, the graphitic nature of PAN-derived carbons is evaluated based on Raman spectroscopy, a standard nondestructive analysis tool. As shown in Fig. 2a, we observe the *D* and *G* peaks centered at approximately 1350 and 1590 cm^−1^, respectively. The spectrum of pure graphite shows a strong *G*-peak due to the in-plane bond-stretching motion of pairs of *C sp*^2^-bonded atoms, while the *D*-peak is more pronounced in the presence of defects such as bond-angle disorder, bond-length disorder, vacancies, and edge defects^28^. The intensity ratio of the *D*- and *G*-peaks (*I*_D_/*I*_G_) is proportional to the in-plane correlation length and corresponds to the amount of disorder present in the carbon wire^29^. The lower the value of *I*_D_/*I*_G_ is, the higher the crystalline microstructure or the lower the disordered (amorphous) nature of the carbon.

Because of the disordered arrangement of carbon wires (Fig. 2a), the tensile force during the carbonization process does not effectively stretch the fibers along the longitudinal direction, resulting in a high-intensity ratio of 1.04 (Fig. 2a). After aligning the carbon wires along the longitudinal direction (Fig. 2a), the resulting intensity ratio of 0.96 (Fig. 2a) is slightly lower than 1.04, showing a higher crystallinity or a lower amorphous nature. Although the alignment of fibers demonstrates the possibility of developing tension to improve crystallinity, the microstructure of this carbon is still glassy in nature based on the results (Fig. 2a).

The formation of glassy carbon during pyrolysis derives from the curved structures of the penta- and hepta-carbon rings^14^. To implement this alignment process for curved structures, a mechanical treatment that changes axial stress from compressive stress to tensile stress is applied to the PAN fibers for a remarkable increase in the graphitic structure of the resulting carbons. Here, the deformation introduced by aligning the polymer nanofibers on a silica surface (Fig. 2a) plays a somewhat similar role to that of mechanical treatment, as described by Maziar et al.^14^. The resulting strong attachment of the polymeric nanofiber to the silicon substrate limits radial shrinkage during stabilization and carbonization, thus forming tension stress in the axial and radial directions. Upon increasing the rotational speed of the drum, the bond of the nanofiber to the silica surface becomes strong enough to counterbalance the tension developed during pyrolysis. Atomic force microscopy allows for a detailed study of the resulting carbon wire microstructure. Visual examination of the atomic-resolution micrographs illustrates the evolution of the developed carbon wire microstructures. Since these two forces have opposite effects on the radial shrinkage of fibers, polymer fibers are transformed into nanograin-based carbon wires (AFM phase images on the left side of Fig. 2a). The resulting intensity ratio of 1.38 is much higher than 0.96 and 1.04, thus poorly crystalline carbon wires are formed.

Nitrogen pretreatment is performed by placing the silicon chip with the carbon dioxide surface (left side of Fig. 2b) in a quartz glass tube filled with nitrogen for heat treatment at 1000 °C to dramatically overcome the breaking of fiber into nanograins during pyrolysis. Once the polymer fibers are deposited on the silica surface pretreated with nitrogen, carbonization transforms the polymer fiber structure into a defect-free carbon wire (AFM phase images in the middle of Fig. 2b). The transition from a nanograin-based microstructure to a defect-free microstructure is accompanied by a decrease in the *I*_D_/*I*_G_ ratio from 1.38 to 0.74 (Fig. 2a, b), thus forming highly crystalline carbon wires. A previous study showed that the graphitic structure in carbon wires showed a remarkable increase with a decrease in the *I*_D_/*I*_G_ ratio from 1.26 to 0.69^14^. The highly crystalline carbon wires are believed to have a much more graphitic structure.

### Effect of nanowire diameter on crystalline structuring

From Fig. 2c, d, it is clear that stress management is crucial to yield highly crystalline carbon nanowires. The tensile stress on the cross-section of the fibers becomes more pronounced with decreasing diameters of the fibers, so we also exploited thinning the wires to further improve the graphitic structure. The effect of the nanowire diameter, which is dependent on the applied voltage in Fig. 1d, on the microstructure and crystallinity of the carbon nanowires on the silica surface pretreated with nitrogen is illustrated in Fig. 2c, d. Further investigation into the decrease in wire diameter from ~5.05 μm to ~45 nm (Fig. 2c, d) reveals an evolution from a porous microstructure to a highly crystalline nanostructure via nanocrystalline structuring. A Raman spectrometer with a DXR microscope and equipped with a 532 nm excitation laser was selected to evaluate the crystallinity of the microstructures in the carbon nanowires fabricated at 1200, 500, and 35 V. The intensity ratio of 0.78 in Fig. 2d is much lower than that of 1.41 in Fig. 2d, corresponding to the higher degree of crystallinity of the nanocrystalline microstructure. This improvement in crystallinity may be attributed to the increase in tensile stress in the axial direction due to the decrease in diameter from ~5.05 μm to ~305 nm (Fig. 2c). Upon further reduction of the carbon nanowire diameter from ~305 nm to ~45 nm (Fig. 2c), the microstructure is transformed into an even more crystalline microstructure, which is gleaned from the decrease in *I*_D_/*I*_G_ from 0.78 to 0.64 (Fig. 2d). Figure 2d illustrates the correlation between the crystallinity of the microstructures and the applied voltage. Clearly, the polymer fibers obtained at the lowest voltages result—after pyrolysis—in the highly crystalline carbon nanowires owing to their highly graphitized structures.

### Effect of decyanation on crystalline structuring

**Fig. 3:**
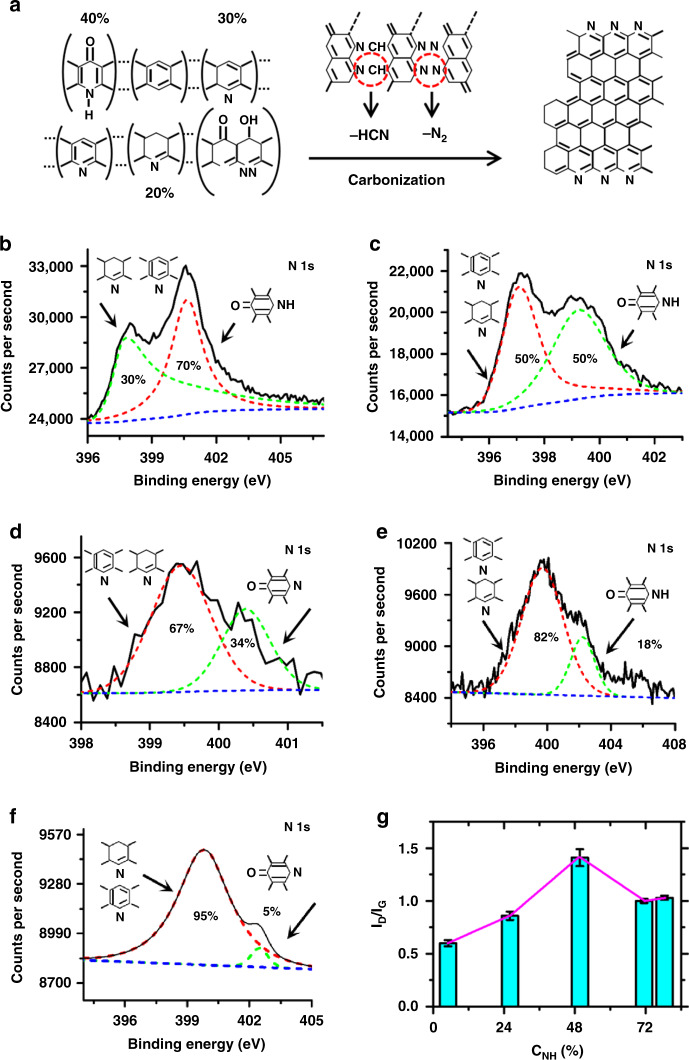
Effect of the decyanation reaction on the crystalline structuring of wires. **a** Model reaction path from stabilized PAN to carbon. N 1 s X-ray photoelectron spectroscopy (XPS) spectra of **b** disordered carbon wires at 1200 V and of arrayed carbon nanowires at **c** 1200 V, **d** 500 V, and **f** 35 V with pretreatment of the silica surface in a nitrogen environment at 1000 °C. **e** N 1 s XPS spectra of arrayed carbon nanowires at 35 V in the absence of nitrogen pretreatment of the silica surface. **g** *I*_D_/*I*_G_ as a function of the intensity of the acridine rings (C_NH_); *n* > 5

The effect of tension stress on crystalline structuring is accompanied by decyanation reactions (named stress decyanation). A previous report showed that relying on denitrogenation and decyanation reactions to remove impurity elements during carbonization (Fig. 3a) resulted in the formation of carbon sheet-like layers from carbon-like ribbons^2^. To correlate the influence of the denitrogenation and decyanation reactions with the graphitic structure, Raman spectroscopy, and X-ray photoelectron spectroscopy characterization was performed. The full N 1 s spectra presented in Fig. 3b–f demonstrate two typical peaks that are assigned to nitrogen atoms in the acridine ring and nitrogen atoms in the naphthyridine and hydronaphthyridine rings. For the porous microstructure of carbon wires with a diameter of ~4.3 µm (Fig. 2c), the corresponding N 1 s spectrum in Fig. 3c has two components, which are assigned to nitrogens in the acridine ring bonds (50%) and in the naphthyridine and hydronaphthyridine rings (50%). In comparison with that of carbon wires fabricated at 1200 V (Fig. 2a), the intensity of the acridine rings is weakened from 70% (Fig. 3b) to 50%, revealing a more extensive decyanation reaction for the carbon wires in an array fabricated at 1200 V. With copious decyanation, most of the reaction products that leave the carbonizing fiber are in the form of gases, including HCN, H_2_O, O_2_, H_2_, CO, NH_3_, and CH_4_^2^, ultimately leading to a porous microstructure. The resulting intensity ratio of 1.41 (Fig. 2d) is much higher than the 0.96 in Fig. 2a but is comparable to the 1.38 from nanograin-based structuring, demonstrating that defects in the carbon wire reduce the degree of crystallization.

After the decrease in the nitrogen intensity of the acridine ring from 34 to 5% (Fig. 3d, f), the resulting intensity ratio (*I*_D_/*I*_G_) decreases from 0.78 to 0.64 (Fig. 2d), demonstrating the possibility of enhancing the decyanation reactions to improve the crystallinity. In the absence of nitrogen pretreatment of the silica surface, the resulting increase in the nitrogen intensity of the acridine ring from 5 to 18% (Fig. 3e, f) demonstrates that the presence of nitrogen at the surface facilitates more extensive decyanation reactions during carbonization, which leads to the improved crystallinity of carbon wire; this is similar to the results shown in Fig. 2a, b. Regardless of the porous defects in Fig. 2c, the effect of *I*_D_/*I*_G_ on the intensity of the acridine rings indeed reveals the improved crystallinity of the carbon wire with more extensive decyanation reactions, as shown in Fig. 3g.

The effect of nanowire diameter and decyanation on crystalline structuring reveals further insight into the underlying mechanism of graphitization at relatively low temperatures (1000 °C). Several previous studies have demonstrated that enhancing the degree of graphitization can, to a large extent, improve the physical and chemical nature of carbon nanowires^14^. Thus, the improvement in crystallinity with extensive decyanation reactions provides an attractive pathway for tailoring the effect of a crystalline surface structure on electrochemical properties.

### Electrochemical characterization using cyclic voltammetry

**Fig. 4:**
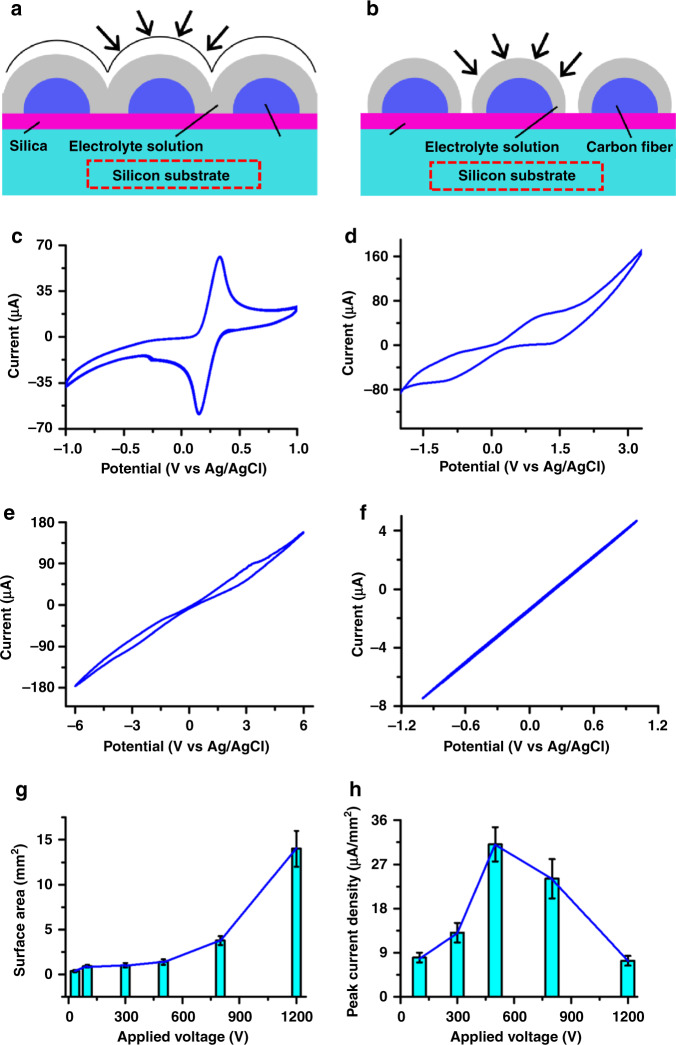
Electrochemical characterization of crystalline carbon nanowire arrays based on cyclic voltammetry. Schematics showing the diffusion of carbon nanowires in the case of **a** overlapping and **b** nonoverlapping diffusion hemispheres. Cyclic voltammetry (CV) curves of 5 mM K_4_Fe(CN)_6_/5 mM K_3_Fe(CN)_6_ in 0.1 M phosphate buffer (pH 7.4) (1:1 mixture) for carbon wire mats disordered at **c** 1200 V and arrayed at **d** 1200 V, **e** 800 V, and **f** 35 V, respectively; *n* > 8. **g** Surface area and **h** peak current density of a carbon nanowire array as functions of applied voltage during NFES; *n* > 8

In the case of very long experimental and theoretical time scales, hemispherical diffusion to microdisk electrodes^30–33^ shows diffusion regimes depending on the CV scan rate or the EIS frequencies. Increasing the frequency to the characteristic value *ω* = *D*/*d*^2^, where *D* is the diffusion coefficient of the analyte and *d* is the distance between neighboring electrodes^34,35^, causes the transition from overlapping (Fig. 4a) to nonoverlapping (Fig. 4b) diffusion hemispheres. By further increasing the frequency to the characteristic value *ω* = *D*/*a*^2^, where *a* is the radius of a single disc electrode, a planar diffusion regime is obtained; thus, a single electrode can be taken as a planar electrode. In the interval given by these two characteristic frequencies, the electrode array shows steady-state behavior with a sigmoidal cyclic voltammetry curve.

**Fig. 5:**
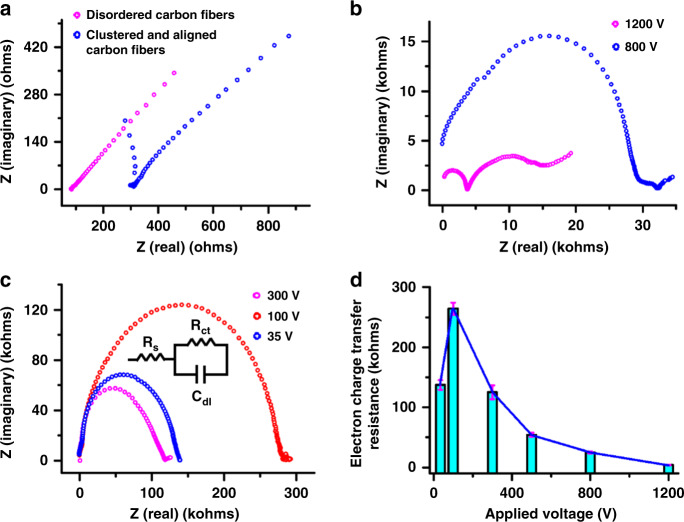
Electrochemical characterization of crystalline carbon nanowire arrays based on impedance spectroscopy. Impedance spectra of the **a** disordered carbon mat at 1200 V and clustered and aligned carbon fiber mat at 1200 V along with the near-field carbon fiber arrays at **b** 1200 and 800 V and **c** at 300, 100, and 35 V. *R*_s_, *R*_ct_, and *C*_dl_ in **c** represent the solution resistance, electron transfer resistance, and double layer capacitance, respectively. AC amplitudes of 1000 mV are applied to the carbon nanofibers in (**a**–**c**). Impedance spectra of near-field carbon fiber arrays at 35 V for AC amplitudes of 1000 and 25 mV; *n* > 6. **d** Electron charge transfer resistance as a function of the applied voltage during NFES; *n* > 10

Cyclic voltammetry (CV) curves of the ferri/ferrocyanide ([Fe(CN)_6_]^3−/4−^) redox couple in aqueous solutions have often been used as a reference method to evaluate the electrochemical performance of a carbon electrode^20,36,37^. The CV curve from the disordered carbon wire mat in Fig. 2a shows macroelectrode behavior with anodic and cathodic peak currents in Fig. 4c, indicating partially overlapping diffusion hemispheres. Aligning the disordered carbon wires to a certain distance (~2.5 μm) at 1200 V results in a typical steady-state sigmoidal voltammogram with a steady-state current in Fig. 4d. This demonstrates the dependency of the nonoverlapping-to-overlapping diffusion regime on the distance of carbon wires in the characteristic frequency of *ω* = *D*/*d*^2^. By reducing the carbon wire diameter further from ~4.3 µm to ~512 nm, the interwire spacing further widens, leading to a decrease in the characteristic frequency (*ω* = *D*/*d*^2^). The resulting CV curve in Fig. 4e still shows steady-state behavior but has a higher steady-state current compared to that of carbon wires arrayed at 1200 V, which is likely derived from increasing the resistance of the carbon wire due to the decrease in diameter. Theoretical and experimental work^30^ on the diffusion of microelectrode arrays show that, in the case of a scan rate of 100 mV/s for *d* ≥ 100*a*, the dominating mode of diffusion is determined by the transition between the planar and hemispherical diffusion layers, leading to a sigmoidal shape in the CV curve. Clearly, the transition from the overlapping of the individual diffusion layers to planar diffusion layers over the entire electrode array in Fig. 4f is very distinct for a highly crystalline carbon nanowire array. The weakening of peak currents in the CV shows a monotonically increasing straight line (Fig. 4f), which is expected due to the large resistance in the thin carbon nanowires (Fig. 5d).

Lowering the applied voltage during NFES increases the specific surface area of carbon nanowires and subsequently improves the degree of graphitization, thus influencing the peak currents of carbon wires, as shown in Fig. 4h. The nanocrystalline-structured wires at 500 V clearly display at least a fourfold higher current than the porous-structured microwires at 1200 V and the highly crystalline nanowires at 35 V. The higher surface area derived from the appearance of the nanocrystalline structure on the surface of the carbon nanowires is well correlated with the highest peak current of the carbon nanowire arrays at 500 V. The highly crystalline carbon nanowire array at 100 V shows the smallest surface area in Fig. 4g but has a peak current equivalent to that of the carbon nanowire array at 1200 V (Fig. 4h). This is most likely due to the highly graphitized structure increasing the electrical conductivity^14^.

### Electrochemical characterization using impedance spectroscopy

Electrochemical impedance spectroscopy (EIS) of the carbon nanowire array was performed using a solution of 5 mM K_4_Fe(CN)_6_/5 mM K_3_Fe(CN)_6_ in a 0.1 M phosphate buffer (pH 7.4). Impedance spectra were obtained at frequencies from 0.1 Hz to 1000 kHz and at a 0 V dc potential vs. Ag/AgCl. The impedance spectra of the disordered carbon wire mat and clustered and aligned carbon wire mat are shown in Fig. 5a. Monotonically increasing straight lines with similar slopes at low frequencies are observed, reflecting a diffusion-limited process with the overlapping diffusion fields of all the participating wires. Upon the formation of the wire-to-wire distance derived from clustered and aligned carbon wires in Fig. 2a, the impedance spectroscopy in Fig. 5a shows a quarter circle at high frequencies and a straight line at low frequencies. This is due to the weakening of the diffusion gradients (*f*) according to Eq. (3):^34^

3 $$f = \frac{{4\tilde \alpha }}{{\pi d^2}}$$

where *f* is the diffusion gradient in front of each individual –ultramicroelectrode and *ᾶ* denotes the arithmetical average of the oxidation and reduction diffusion coefficients of [Fe(CN)_6_]^3−/4−^.

By arraying all the carbon nanowires and maintaining a wire-to-wire spacing of ~2.5 μm, the impedance spectrum shows two semicircles connected together at high frequencies and a relatively short straight line with a similar slope (diffusion limitation) at the lowest frequencies, as shown in Fig. 5b. Theoretical and experimental work^34^ on the impedance of ultramicroelectrode arrays exhibit a semicircle at low frequencies in addition to the well-known semicircle at higher frequencies, demonstrating overlap of the diffusion gradients. The change from overlapping diffusion to diffusion limitation is shown for carbon nanowire arrays at 1200 V. This demonstrates the weakening of the overlap of the diffusion gradients compared to that of the clustered and aligned carbon wires.

By reducing the applied voltage from 1200 to 800 V, which is accompanied by a decrease in the wire diameter from ~4.3 µm to ~512 nm, the impedance spectrum changes to a large semicircle with a small straight line, as shown in Fig. 5b. The semicircle diameter at higher frequencies is governed by the charge transfer resistance (*R*_et_) related to the electron transfer rate of the redox species on the electrode surface. The region at low frequencies again represents a line with a similar slope, showing mass transfer control or a diffusion limitation. Previously, in the case of three-dimensional hemispherical diffusion to an array of microdisk electrodes^31–34^, the impedance spectrum showed a semicircle for frequencies between the characteristic frequencies of *ω* = *D*/*d*^2^ and *ω* = *D*/*a*^2^. The transition from overlapping diffusion to three-dimensional hemispherical diffusion at high frequencies is shown to observe the changes between the impedance spectra of carbon nanowire arrays at 1200 and at 800 V. The impedance spectrum of the carbon nanowire array at 800 V is expected due to the lower density of arrayed carbon wire^20^, which is derived from widening the wire-to-wire distance with decreasing wire diameter.

Reducing the diameter of the arrayed carbon wire from ~512 to ~40 nm with decreasing applied voltage from 800 to 35 V results in changes in the impedance spectrum from a large semicircle with a small straight line to just a single semicircle without a Warburg impedance element (Fig. 5c). This case allows for the process from the overlapping diffusion of all the participating wires to the complete reaction kinetics determined by electron transfer. Theoretical work^35^ has shown that a smaller electrode radius leads to a larger electrode admittance per unit area, which allows the susceptance at low frequencies to remain negligible. In addition, decreasing the nanowire diameter increases the lower frequency limit at which the susceptance (imaginary part of the impedance) becomes lower than the conductance (real part of the impedance). Thus, the impedance spectrum of the highly crystalline carbon nanowire array at 35 V shows a semicircle for *ω* = *D*/*a*^2^ *» D*/*d*^2^. These results demonstrate the ability to control the diffusion regimes by altering the wire spacing and nanowire size.

In most studies, the semicircle spectrum from carbon nanowire arrays is meaningful only when ac signals with amplitudes ≤25 mV are applied^38,39^. By reducing the amplitude from 1000 mV to 25 mV, the impedance spectrum of the highly crystalline carbon nanowires in Fig. 5c still shows a single semicircle without scattered data points, demonstrating the high signal-to-noise ratio compared to that of typical carbon nanowire electrode arrays^20^. Therefore, highly crystalline carbon nanowire arrays can be selected as potential candidates for DNA switching^40^.

The impedance spectra were fitted by means of an equivalent Randles circuit with capacitance, charge transfer resistance, solution resistance, and Warburg elements. The charge transfer resistance related to the overall surface area of the carbon nanowire arrays corresponds to the diameter of the semicircle in the high-frequency domain of the impedance spectra. Figure 5d shows the increase in charge transfer resistance as the applied voltage is decreased from 1200 to 100 V; notably, this is accompanied by a decrease in the wire diameter from ~4.3 µm to ~120 nm and the microstructural transformation from a porous microstructure to a nanocrystalline microstructure. It is well known from theoretical and experimental work on carbon microelectrodes that the smaller the electrode surface area is, the greater the charge transfer resistance^41^. The monotonic correlation between the charge transfer resistance and wire diameter in Fig. 5d is expected in accordance with the results of carbon microelectrodes. Clearly, by changing the wire diameter in the carbon nanowire arrays, the semicircle scale at high frequencies can be adjusted.

A previous study^14^ on the correlation between microstructures and properties showed that enhancing graphitic microstructures could improve the bulk characteristics of carbon fabrics, resulting in an increase in conductivity from ~200 S/m to ~5000 S/m with improving graphitization. The charge transfer resistance of highly crystalline carbon nanowire arrays at 35 V is unusually low and is half of that of carbon nanowire arrays at 100 V (Fig. 5d). This is most likely due to the further improvement of the graphitic microstructure as the voltage is decreased from 100 to 35 V (Fig. 2d). Thus, the combination of these factors, including the wire-to-wire spacing, nanoscale diameter, and graphitic microstructure, leads to a distinct semicircle of highly crystalline carbon nanowire arrays in EIS.

## Conclusion

In the state-of-the-art fabrication of carbon nanowires with the carbon-nano-electro-mechanical system approach, the growth of a graphitic microstructure is typically limited to the use of a Ni catalyst. In this work, we demonstrated a novel catalyst-free fabrication process for arrays of highly graphitized carbon nanowires with various surface nanostructures. It was developed by minimizing the polymer fiber diameter with ultralow-voltage NFES. The linear speed of the spinneret and the rotational speed of the collector allowed for better control of the fiber-to-fiber distance in the fiber arrays. The stabilization at 115 °C and subsequent carbonization converted these polymer nanofibers arrayed on a silica surface into carbon nanowires. The thickness control of carbon nanowires was conveniently adjusted by tuning the applied voltage during NFES. Aligning the wires on a nitrogen-pretreated silica surface allowed for stress decyanation to improve the graphitic content of the carbon nanowires. The presented diameter control showed the ability to transform a porous-microstructured carbon wire into a highly crystalline nanostructure via nanocrystalline structuring.

Various electrochemical behaviors were observed in the obtained CV curves when reducing the wire diameter because that altered the structure on the surface of the carbon wire and improved the graphitic structure. The typical CV curve showing macroelectrode behavior with anodic and cathodic peak currents was altered to a sigmoidal CV curve with the steady-state current dominated by radial diffusion. This is a characteristic behavior observed for carbon nanowire arrays where there is no overlap of the diffusion hemispheres from neighboring electrodes. Compared to three-dimensional carbon nanowire electrode arrays with wire diameters of ~100 nm, two-dimensional carbon nanowire arrays with diameters of ~512 nm easily obtained low steady-state currents during cyclic voltammetry, which has been regarded as a potential advantage for biosensing applications. –The nanocrystalline structure on the surface of graphitized carbon wires rendered extremely high peak currents in their CV curve. More importantly, highly crystalline carbon nanowire arrays showed a linear CV curve without anodic and cathodic peak currents, representing the special characteristics of the highly graphitized structures in the nanoscale carbon wires. The charge transfer resistance could be controlled by varying the diameter of the carbon nanowires. In the EIS spectra of the highly crystalline carbon nanowire arrays, the abnormal decrease in the charge transfer resistance confirmed the influence of the graphitized structure on the electrochemical behavior.

The electrochemical performance of the crystalline carbon nanowire arrays with various surface nanostructures makes them potential candidates for biochemical sensors with lower detection limits and as devices for electrochemical energy storage. Graphitized carbon submicrowires with a nanocrystalline structure could allow for amplified biosensing via redox cycling and enhanced capacitive energy storage in microsupercapacitors. The linear CV curve of highly crystalline carbon nanowires shows the ability to permit stable data collection for ultrasensitive biological detection. In particular, a further increase in the surface area of –nanocrystalline-structured carbon wires could allow for high-power supercapacitors.

## Materials and methods

The electrospinning solution (9% PAN) was prepared by dissolving PAN (150000 mw, Sigma Aldrich, St. Louis, MO) in DMF. Using vortex mixing at 30 RPM, mixtures of PAN/DMF were allowed to freely diffuse at different temperatures. For the NFES experiments, we used a 3 mL syringe mounted on a syringe pump to dispense the highest conductivity ink at a feed rate below 10 nL/min. Silicon substrates coated with silicon dioxide were mounted in the grooves and convex areas on the drum with carbon tape. The NFES voltage was applied between the dispensing needle and the grounded drum. It should be noted that regular arrays were achieved as long as *ω* ≥ 400 RPM and *ν* ≥ 80 µm/s were achieved at the same time. Fabrication of carbon nanowire arrays derived from PAN nanofiber arrays consisted of oxidization in the air at 115 °C (named stabilization) and subsequent carbonization in a furnace with an inert nitrogen atmosphere at 1000 °C (heating ramp rate of 15 °C/min) (Fig. 1b).

In general, the graphitic nature of PAN-derived carbons was evaluated based on Raman spectroscopy, a standard nondestructive analysis tool. The Raman spectrometer with a DXR microscope (Thermo Fisher) and equipped with a 532 nm excitation laser assessed the corresponding carbon wire arrays. By moving the platform on which the sample was located, the wire was moved to a location just below the marked laser spot. Linear scanning was implemented with a step size of 100 nanometers. For each step, data were collected by exciting the laser, ultimately resulting in obtaining the Raman spectrum of a carbon nanowire. We used the lens for an average of approximately 20 times per sample. To correlate the influence of denitrogenation and the decyanation reaction on the graphitic structure, X-ray photoelectron spectroscopy was performed.

Atomic force microscopy (AFM) (Bruker Dimension Icon) and scanning electron microscopy (SU8220) allowed for a detailed study of the surface nanostructures in the resulting carbon fibers. The topology of carbon fibers was studied with the tapping mode to investigate the influence of the applied voltage on various surface nanostructures.

A traditional three-electrode configuration consisting of an Ag/AgCl reference electrode, Pt counter electrode, and carbon working electrode was used. To probe the carbon/electrolyte interface, a GPSTAT12 potentiostat/galvanostat equipped with a frequency response analyzer module was employed. Cyclic voltammetry (CV) of the ferri/ferrocyanide ([Fe(CN)_6_]^3−/4−^) redox couple in aqueous solutions have often been used as a reference method to evaluate the electrochemical performance of a carbon electrode^20,36,37^.

Electrochemical impedance spectroscopy (EIS) of the carbon nanofiber array was performed using a solution containing 5 mM K_4_Fe(CN)_6_/5 mM K_3_Fe(CN)_6_ in a 0.1 M phosphate buffer (pH 7.4). Impedance spectra were taken at frequencies from 0.1 Hz to 1000 kHz and at a dc potential of 0 V vs. Ag/AgCl. The number of experiments (*n*) is indicated in the figure legends.
